# Spike Timing Dependent Plasticity Finds the Start of Repeating Patterns in Continuous Spike Trains

**DOI:** 10.1371/journal.pone.0001377

**Published:** 2008-01-02

**Authors:** Timothée Masquelier, Rudy Guyonneau, Simon J. Thorpe

**Affiliations:** 1 Centre de Recherche Cerveau et Cognition, Université Toulouse 3, Centre National de la Recherche Scientifique (CNRS), Faculté de Médecine de Rangueil, Toulouse, France; 2 SpikeNet Technology SARL, Prologue 1 La Pyrénéenne, Labège, France; Indiana University, United States of America

## Abstract

Experimental studies have observed Long Term synaptic Potentiation (LTP) when a presynaptic neuron fires shortly before a postsynaptic neuron, and Long Term Depression (LTD) when the presynaptic neuron fires shortly after, a phenomenon known as Spike Timing Dependant Plasticity (STDP). When a neuron is presented successively with discrete volleys of input spikes STDP has been shown to learn ‘early spike patterns’, that is to concentrate synaptic weights on afferents that consistently fire early, with the result that the postsynaptic spike latency decreases, until it reaches a minimal and stable value. Here, we show that these results still stand in a continuous regime where afferents fire continuously with a constant population rate. As such, STDP is able to solve a very difficult computational problem: to localize a repeating spatio-temporal spike pattern embedded in equally dense ‘distractor’ spike trains. STDP thus enables some form of temporal coding, even in the absence of an explicit time reference. Given that the mechanism exposed here is simple and cheap it is hard to believe that the brain did not evolve to use it.

## Introduction

**Figure 1:**
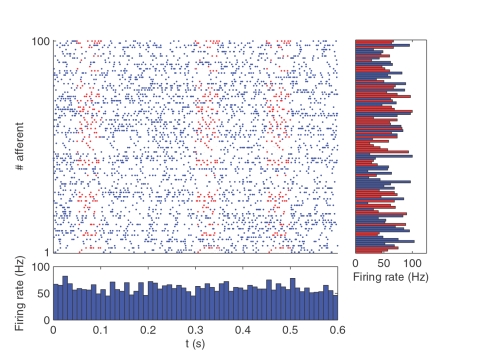
Spatio-temporal spike pattern. Here we show in red a repeating 50 ms long pattern that concerns 50 afferents among 100. The bottom panel plots the population-averaged firing rates over 10 ms time bins (we chose 10 ms because it is the membrane time constant of the neuron used later in the simulations), and demonstrates that nothing characterizes the periods when the pattern is present. The right panel plots the individual firing rates averaged over the whole period. Neurons involved in the pattern are shown in red. Again, nothing characterizes them in terms of firing rates. Detecting the pattern thus requires taking the spike times into account.

Electrophysiologists report the existence of repeating spatio-temporal spike patterns with millisecond precision, both in vitro and in vivo, lasting from a few tens of ms to several seconds[1]–[3]. In this study we assess the difficult problem of detecting them, and suggest how neurons could solve it. The problem is made particularly difficult when only a fraction of the recorded neurons are involved in the pattern. Fig. 1 illustrates such a situation. There is a pattern of spikes (indicated by the red dots) that repeats at irregular intervals, but is hidden within the variable background firing of the whole population (shown in blue). The problem is made hard because nothing in terms of population firing rate characterizes the periods when the pattern is present, nor is there anything unusual about the firing rates of the neurons involved in the pattern. In such a situation detecting the pattern clearly requires taking the spike times into account. However direct comparison of each spike time to one another over the entire recording period and across the entire set of afferents is extremely computationally expensive. In this article we will see how a single neuron equipped with STDP can solve the problem in a different manner, taking advantage of the fact that a pattern is a succession of spike coincidences.

**Figure 2:**
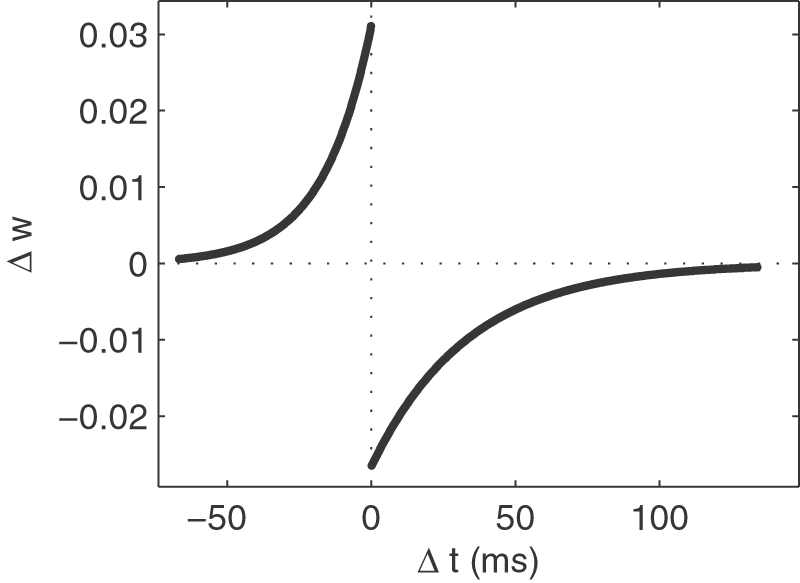
The STDP modification function. We plotted the additive weight updates as a function of the difference between the presynaptic spike time and the postsynaptic one. We used an exponential law (see Materials and Methods). The left part corresponds to Long Term Potentiation (LTP) and the right part to Long Term Depression (LTD).

STDP is now a widely accepted physiological mechanism of activity-driven synaptic regulation. It has been observed extensively in vitro[4]–[7], and more recently in vivo in Xenopus's visual system[8], [9], in the locust's mushroom body[10], and in the rat's visual cortex[11] and barrel cortex[12]. An exponential update rule fits well the synaptic modifications observed experimentally[13] (see Fig. 2). Very recently, it has also been shown that cortical reorganization in cat primary visual cortex is in accordance with STDP[14]. Note that STDP is in agreement with Hebb's postulate because it reinforces the connections with the presynaptic neurons that fired slightly before the postsynaptic neuron, which are those that ‘took part in firing it’. It thereby reinforces causality links.

When a neuron is presented successively with similar volleys of input spikes STDP is known to have the effect of concentrating synaptic weights on afferents that consistently fire early, with the result that the postsynaptic spike latency decreases[15]–[18]. This theoretical observation is in accordance with recordings in rat's hippocampus showing that the so called ‘place cells’ fire earlier – relative to the cycle of the theta oscillation in hippocampus – after the animal has repeatedly traversed the corresponding area[19]. STDP has also been studied in an oscillatory mode, and was shown to be able to select only phase-locked inputs among a broad population with random phases, turning the postsynaptic neuron into a coincidence detector[20].

The main limitation of these studies is the assumption that the input spikes arrive in discrete volleys (sometimes also called ‘spike waves’). They assume an explicit time reference – usually the presentation of a stimulus[15], [17], [18], or the maximum (or minimum) of an oscillatory drive[20], [21] – that allows the specification of a time-to-first spike (or latency) for the afferents, which could be used by the brain to encode information[22], [23]. Activity between the volleys is assumed to be spontaneous and much weaker. Furthermore, many studies[15], [17], [20] also require the pattern to be present in all volleys for the STDP to learn it, that is no ‘distractor’ volleys are inserted between pattern presentations. But what happens when the population of afferents is continuously firing with a constant population firing rate, so that no explicit time reference is available? Is STDP still able to find and learn spike patterns among the inputs? Is the learning robust if, more realistically, pattern presentations occur at unpredictable times, separated by long ‘distractor’ periods and if the pattern does not involve all the afferents? Does it make sense to use the beginning of the pattern as a time reference, and does the postsynaptic spike latency with respect to this reference still decrease?

**Figure 3:**
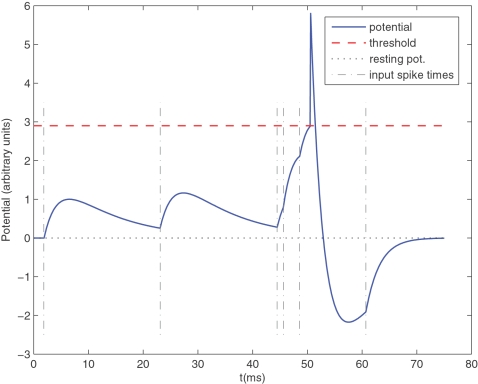
Leaky Integrate-and-Fire (LIF) neuron. Here is an illustrative example with only 6 input spikes. The graph plots the membrane potential as a function of time, and clearly demonstrates the effects of the 6 corresponding Excitatory PostSynaptic Potentials (EPSP). Because of the leak, for the threshold to be reached the input spikes need to be nearly synchronous. The LIF neuron is thus acting as a coincidence detector. When the threshold is reached, a postsynaptic spike is fired. This is followed by a refractory period of 1 ms and a negative spike-afterpotential.

To answer these questions we inserted an arbitrary pattern at various times into randomly generated ‘distractor’ spike trains, as in Fig 1, and investigated whether a single receiving STDP neuron, with a 10 ms membrane time constant, was able to learn it in an unsupervised manner. To be precise, we simulated a population of 2,000 afferents firing continuously for 450 s (see Materials and Methods for details). Most of the time (3/4 of the time in the baseline simulation) the afferents fired according to a Poisson process with variable instantaneous firing rates. Spiking activity in the brain is usually assumed to follow roughly Poisson statistics, hence this choice, but here it is not crucial: what matters is that the afferents fire stochastically and independently. But every now and then, at random times, half of these afferents left the stochastic mode for 50 ms and adopted a precise firing pattern. This repeated pattern had roughly the same spike density as the stochastic distractor part, so as to make it invisible in terms of firing rates. To be precise the firing rate averaged over the population and estimated over 10 ms time bins has a mean of 64 Hz and a standard deviation of less than 2 Hz (this firing rate is even more constant than in the 100 afferent case of Fig. 1 because of the law of large numbers). We further increased the difficulty by adding a permanent 10 Hz Poissonian spontaneous activity to all the neurons, and by adding a 1 ms jitter to the pattern. Intriguingly, we will see that one single Leaky Integrate-and-Fire (LIF) neuron receiving inputs from all the afferents, acting as a coincidence detector (see Fig. 3), and implementing STDP, is perfectly able to solve the problem and learns to respond selectively to the start of the repeating pattern.

## Results

**Figure 4:**
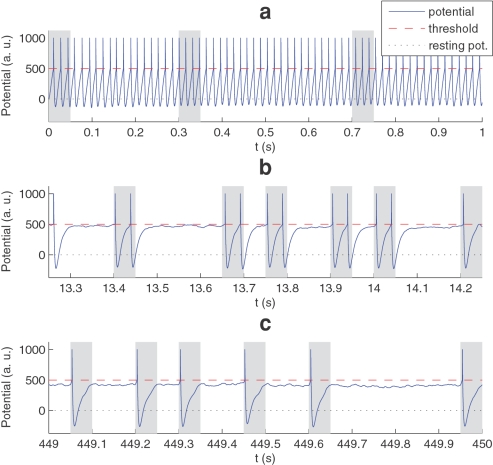
Overview of the 450 s simulation. Here we plotted the membrane potential as a function of simulation time, at the beginning, middle, and end of the simulation. Grey rectangles indicate pattern presentations. (a) At the beginning of the simulation the neuron is non-selective because the synaptic weights are all equal. It thus fires periodically, both inside and outside the pattern. (b) At t≈13.5 s, after about 70 pattern presentations and 700 discharges, selectivity to the pattern is emerging: gradually the neuron almost stops discharging outside the pattern (no false alarms), while it does discharge most of the time the pattern is present (high hit rate), here even twice (c) End of the simulation. The system has converged (by saturation). Postsynaptic spike latency is about 4 ms. Hit rate is 99.1% with no false alarms (estimated on the last 150 s).

At the beginning of a first simulation the 2,000 synaptic weights are all equal to 0.475 (arbitrary units normalized in the range [0,1]). The neuron is therefore non-selective. Since the presynaptic spike density – on its 10 ms time scale – is almost constant, it discharges periodically (see Fig. 4a). The greater are the initial weights (or the lower the threshold), the smaller is the period (here it is about 16 ms, the initial firing rate is thus about 63 Hz). Each time a discharge occurs we update the synaptic weights using the STDP rule of Fig. 2, and clip them in the range [0,1]. At this stage, the neuron discharges both outside and inside the pattern (represented by grey rectangles on Fig. 4). In the first case presynaptic and postsynaptic spike times are uncorrelated, and since *a* ^−^ *τ* ^−^>*a* ^+^ *τ* ^+^ (where *a* ^−^ and *τ* ^−^ are respectively the LTD learning rate and time constant, and *a* ^+^ and *τ* ^+^ are the same parameters for LTP, see Materials and Methods), STDP leads to an overall weakening of synapses[15] (note: if no repeating patterns were inserted STDP would thus gradually decrease the synaptic weights until the threshold would not be reached any longer). But in the second case, by reinforcing the synaptic connections with the afferents that took part in firing the neuron, STDP increases the probability that the neuron fires again next time the pattern is presented (reinforcement of causality link). As a result, selectivity to the pattern emerges, here after about 13.5 s (see Fig. 4b) that is after only about 70 pattern presentations and 700 discharges: the neuron gradually stops discharging outside the pattern (no false alarms), while it does discharge most of the time when the pattern is presented (high hit rate), and can even fire twice per pattern as in the case illustrated here. Chance determines which part(s) of the pattern the neuron becomes selective to at this stage (i.e. the postsynaptic spike latency(ies), with respect to the beginning of the pattern here about 5 ms and 40 ms). However the increase in selectivity usually rapidly leads to only one discharge per pattern, here at about 40 ms.

Once selectivity to the pattern has emerged STDP has another major effect. Each time the neuron discharges in the pattern, it reinforces the connections with the presynaptic neurons that fired slightly before in the pattern. As a result next time the pattern is presented the neuron is not only more likely to discharge to it, but it will also tend to discharge earlier. In other words, the postsynaptic spike latency locks itself to the pattern and decreases steadily (with respect to the beginning of the pattern). However, it cannot decrease endlessly. There is a convergence by saturation when all the spikes in the pattern that precede the postsynaptic spike already correspond to maximally potentiated synapses, and all are necessary to reach the threshold. This usually occurs when the latency is already very short, the value depending on the threshold, although it could occur even earlier if the pattern has a zone with low spike density. Spikes outside the pattern cannot contribute efficiently to the membrane potential: since their times are stochastic, STDP usually depresses the corresponding synapses. We end up with a bimodal weight distribution with synapses either maximally potentiated or fully depressed (as predicted by van Rossum et al[24]).

Here this convergence occurs after about 2000 discharges. At this stage, the postsynaptic spike latency (with respect to the beginning of the pattern) is about 4 ms (see Fig. 4c). After convergence the hit rate is then 99.1% with no false alarms (estimated on the last 150 s). Notice that the signal/noise ratio has increased with respect to the situation in Fig. 4b, that is the potential reached on distractor periods is farther from the threshold. Among the 2,000 synapses, 383 are fully potentiated (weight≈1), while the rest of them are almost completely depressed (weight≈0). All of the potentiated synapses correspond to afferents involved in the pattern. The fact that there is no false alarms means once the learning has been done, a neuron just waits for its preferred stimulus, and need never forget what it has learned. The model thus predicts that fully specified neurons might actually have very low spontaneous rates, whereas higher rates might characterize less well specified cells.

**Figure 5:**
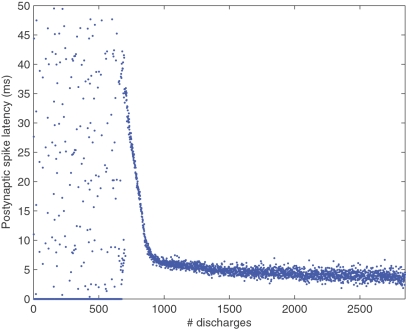
Latency reduction. Here we plotted the postsynaptic latency as a function of the number of discharges (by convention the latency is 0 when the neuron discharged outside the pattern, i.e. when it generated a false alarm). We clearly distinguish 3 periods: the beginning, when the neuron is non-selective; the middle, when selectivity has emerged and STDP is ‘tracking back’ through the pattern; and the end, when the system has converged towards a fast and reliable pattern detector.

Fig. 5 shows the latency reduction (with respect to the beginning of the pattern) during the learning stage until it stabilizes at a minimum of about 4 ms. Apart from the initial part (before selectivity emerges) the curve looks similar to those observed in earlier work with discrete spike volleys[17]. By convention the latency is 0 when the neuron discharged outside the pattern, that is when it generated a false alarm. There are no false alarms after the 676th discharge, that is for the last 436 s of simulation.

**Figure 6:**
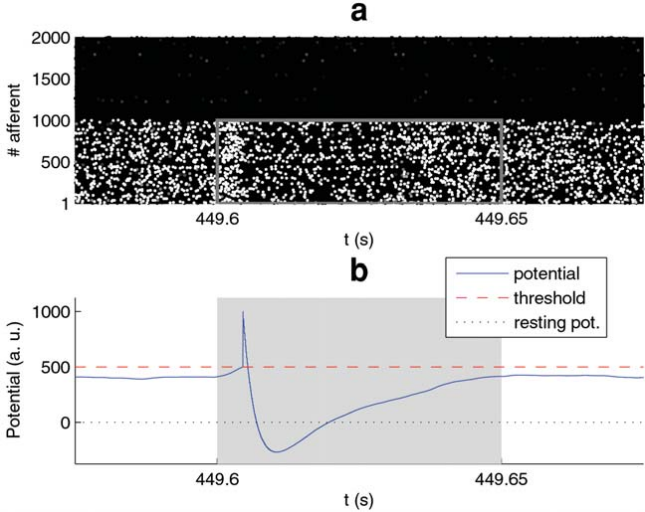
Converged state (a) we represented the spike trains of the 2,000 afferents. We have reordered the afferents with respect to Fig. 1 so that afferents 1–1000 are involved in the pattern, and afferents 1001–2000 are not and we use a color code ranging from black for spikes that correspond to completely depressed synapses (weight = 0) to white for spikes that correspond to maximally potentiated synapses (weight = 1). This allows the visualization of the spikes which generate a significant EPSP and those which do not. The pattern is represented with a grey line rectangle. Notice the cluster of white spikes at the beginning of it: STDP has potentiated most of the synapses that correspond to the earliest spikes of the pattern. Note that virtually all the synaptic connections with afferents not involved in the pattern have been completely depressed. (b) The membrane potential is plotted as a function of time, over the same range as above. We clearly see the sudden increase that corresponds to the above-mentioned cluster.

Fig. 6 illustrates the situation after convergence. It can be seen that STDP has potentiated most of the synapses that correspond to the earliest spikes of the pattern (Fig. 6a), and depressed most of the synapses that correspond to presynaptic spikes which follow the postsynaptic one, as in the previous work with discrete volleys [15], [17], [18]. This results in a sudden increase in membrane potential when the neuron starts integrating the pattern, and the threshold is quickly reached (Fig. 6b). Notice that all the synaptic connections with afferents not involved in the pattern have been completely depressed.

We performed 100 similar simulations with different pseudo-randomly generated spike trains and patterns. Our criteria for a ‘successful’ simulation were: convergence to a state with a postsynaptic latency inferior to 10 ms, a hit rate superior to 98% and no false alarms. This occurred in 96% of the cases. For the remaining 4%, the neurons stopped firing when too many discharges occurred outside the pattern in a row (leading to an overall weakening of synapses, so the threshold was no longer reached).

We ran other batches of 100 simulations to systematically investigate the impact on this 96% success performance of five parameters.

**Figure 7:**
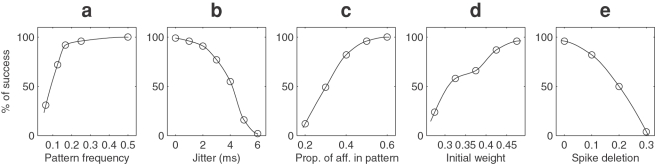
Resistance to degradations (100 trials). (a) Percentage of successful trials as a function of the pattern frequency (pattern duration/the total duration, given a fixed pattern length of 50 ms). The pattern needs to be consistently present, at least at the beginning, for the STDP to start the learning process. (b) Percentage of successful trials as a function of jitter. For jitter greater than 3 ms spike coincidences are lost and the STDP weight updates are inaccurate, so the learning is impaired (c) Percentage of successful trials as a function of the proportion of afferents involved in the pattern. Performance is good if this proportion is above 1/3 (d) Percentage of successful trials as a function of the initial weights. With a high value the neuron can handle more discharges outside the pattern. (e) Percentage of successful trials as a function of the proportion of spikes deleted. With a 10% deletion the pattern was correctly learnt in 82% of the cases.

The first one is the pattern relative frequency (i.e sum of pattern durations over total duration ratio, assuming a fixed pattern duration of 50 ms), 1/4 in the baseline condition, and Fig. 7a shows its effect. We see that while the performance is very high as long as the ratio is above 15%, with smaller values the probability of success drops. This means the pattern needs to be consistently present for the STDP to learn it. However, this applies only at the beginning (say during the first 1000 discharges). Here we used a constant pattern frequency, but after the initial part the neuron has already become selective to the pattern, so presenting longer distractor periods does not perturb the learning at all. We also tried to change the pattern duration while maintaining its relative frequency at 1/4. It turns out that what makes the detection difficult is the delay between two pattern presentations, not the pattern duration itself. Since we kept the pattern relative frequency constant, this delay increased with the pattern duration so the performance dropped: 97% with a 40 ms pattern, 96% with 50 ms, 93% with 60 ms, 59% with 100 ms and 46% with 150 ms. However we think this delay is more naturally investigated by changing the pattern relative frequency as in Fig. 7a.

The second parameter we investigated is the amount of jitter (1 ms in the baseline condition), and Fig. 7b shows its influence. We see that the performance is very good for jitter levels lower than 3 ms. For larger amounts of jitter the spike coincidences are lost, and the STDP weight updates are inaccurate, so the learning is impaired. In the brain millisecond spiking precision has been reported in many structures, including the retina[25], [26], the Lateral Geniculate Nucleus[27], [28], the visual cortex[29], [30], the somatosensory system[31], [32] and the auditory system[33]. Some authors report higher variability, but this could result from non controlled variables rather than intrinsic noise (see Discussion).

The third parameter is the proportion of afferents involved in the pattern (1/2 in the baseline condition), and Fig. 7c shows its influence. The threshold was scaled proportionally. Not surprisingly, with fewer afferents involved in the pattern, it becomes harder to detect, but it is still detected more than half of the times when only 1/3 of the afferents are involved in the pattern. Note that the other 2/3 of afferents are discarded by STDP. This suggests that activity-driven mechanisms could select a small set of ‘interesting’ afferents among a much bigger set of initially connected afferents, probably specified genetically, a phenomenon known as ‘developmental exuberance’ for which there is considerable experimental evidence[34].

The fourth parameter is the initial weight (0.475 in the baseline condition) and Fig. 7d shows its influence. Recall discharges outside the pattern lead to an overall decrease of synaptic weights. If too many of them occur in a row the threshold may no longer be reachable. Thus a high initial value for the weights increases the resistance to discharges outside the pattern, leading to a better performance. High initial weights also cause the neuron to discharge at a high rate at the beginning of the learning process, when it is non-selective: 63 Hz for an initial weight of 0.475, 38 Hz for 0.325. These values may seem high in regard to usual experimental values. But first after only 13 s selectivity has emerged, and the neuron fires at a rate between 5 and 10 Hz. It is conceivable that electrophysiologists rarely record such short very active initial phases. Second, we consider here that the population of afferents is constantly firing with a mean rate of 64Hz. This is to make the problem of pattern detection harder, but if the afferents have less active periods, which is likely to occur in the brain, so will have the post-synaptic neuron. We also added Gaussian noise to the initial weights, with increasing standard deviation until 0.475 (thus equal to the mean). Following this noise addition the weights were clipped in [0,1]. This had no significant impact on the performance, at least in the present case when the initial weights are relatively high.

The fifth parameter is the proportion of missing spikes (0 in the base line condition). The threshold was scaled proportionally. Not surprisingly the number of successfully learned patterns decreases with the proportion of spikes deleted. However with a 10% deletion the pattern was correctly learnt 82% of the time, demonstrating that the system is quite robust to spike deletion.

We also tried changing the membrane time constant *τ_m_* (10 ms in the baseline condition), scaling the threshold proportionally. This had little impact on the performance (79% success with τ*_m_* = 5 ms, 88% with τ*_m_* = 20 ms), but it did have an impact on the minimal latency that is reached after convergence. A smaller time constant (and the smaller threshold that goes with it) causes the neuron to be interested in more coincident spikes. The system converges when the very few nearly coincident first spikes of the pattern all correspond to maximally potentiated synapses, and the postsynaptic spikes is fired just after them. The final latency is thus shorter than the one we have with a longer time constant, which enables the neuron to integrate spikes over a longer time window.

Taken together these results demonstrate that the learning is amazingly robust to the model parameters. We thus believe that we have captured a mechanism than emerges from STDP rather than from a precise neural model configuration. While we admit it is still somewhat speculative to affirm that a similar mechanism takes place in the brain, it is at least very plausible.

## Discussion

Our first claim is that the main results previously obtained for STDP based learning with the highly simplified scheme of discrete spike volleys[15]–[18] still stand in this more challenging continuous framework. This means that global discontinuities such as saccades or micro-saccades in vision and sniffs in olfaction[35], or brain oscillations in general[23] are not necessary for STDP-based learning of temporal patterns (although they will almost certainly help). Temporal code skeptics often point out the fact that neurons would need to know a time reference to decode a temporal code, and we see here that this is not necessary: as long as there are recurrent spike patterns in the inputs, and even if they are embedded in equally dense ‘distractor’ spike trains, a neuron equipped with STDP can potentially find them in only a few tens of pattern presentations, and will gradually respond faster and faster when the pattern is presented, by potentiating synapses that correspond to the earliest spikes of the patterns, and depressing all the others. This last point strongly reinforces the idea that a substantial amount of information could be available very rapidly, in the very first spikes evoked by a stimulus[36].

It is worth mentioning that the proposed learning scheme is fully unsupervised. No teaching signal tells the neuron when to learn nor labels the inputs. Biologically plausible mechanisms for supervised learning of spike patterns have also been proposed[37].

It is also surprising to see how such a simple mechanism can solve a problem as complex as spike pattern detection. However, there is no consensus on the definition of a spike pattern, and we admit ours is quite simple: here a pattern is seen as a succession of coincidences. A Leaky Integrate and Fire (LIF) neuron is known to be capable of coincidence detection, and it has even been proposed that this is its main function in the brain[38], [39]. Here the membrane time constant (10 ms) is shorter than the duration of the pattern (50 ms), and so the LIF neuron can never be selective to the whole pattern. Instead, it is selective to ‘one coincidence’ of the pattern at a time, that is, selective to the nearly simultaneous arrival of certain spikes, just as it occurs in one subdivision of the pattern. At the beginning of the learning process STDP will cause the LIF neuron to become selective to one such coincidence (chance determines which one). Then STDP will track back through the pattern, from one coincidence to the previous one, until the initial coincidence is reached and the chain of causality is stopped. At this point the neuron is selective only to the simultaneous arrival of the pattern's earliest spikes, and can serve as ‘earliest predictor’ of the subsequent spike events[15], [16], [19], at the risk of triggering a false alarm if these subsequent events don't occur, but with the benefit of being very reactive.

This contrasts with approaches where the whole pattern needs to be taken into account, sometimes including finer structural aspects such as spike orders or relative delays[2], [3], [40], [41]. But neuronal mechanisms able to reliably decode such structures have to be proposed and looked for in the brain. One appealing candidate mechanism is the synfire chain[42] but direct evidence for their existence is still fairly limited[43]. Here we limit the notion of pattern to successive coincidences, and suggest a way such patterns could be decoded, using widely accepted neurophysiological mechanisms, namely coincidence detection and STDP.

Another limitation of this work is the excitatory-only scheme. Consequently, something like ‘afferent A must not spike’ cannot be learnt, only ‘positive patterns’ can. However, evidence for plasticity in inhibitory synapses in the brain is weak and inhibition is often assumed to be non-selective. So we propose that most of the selectivity could be achieved using only excitatory synapses, as in this model.

Whether spike times contain additional information with respect to discharge rates has been the object of an ongoing debate for some time. Electrophysiologists have tried to answer this question mostly by recording neurons in sensory and motor systems with a repeating stimulus or action, and looking at inter-trial variability of the spike times. Some claim that spike times can be very reliable while others are more skeptical (see ref [22], [44] for reviews). Given that the simple and cheap mechanism exposed here reliably detects spatio-temporal spike patterns, it is hard to believe that the brain did not evolve to use at least the form of temporal coding exposed above (‘successive coincidences’), unless there is an unavoidable intrinsic source of noise in the integrate-and-fire mechanism that makes all spike times unreliable. The main source for this sort of noise is probably at the level of synaptic transmission[45], since neurons stimulated directly by current injection in the absence of synaptic input give highly stereotyped and precise responses[46]. However, spike times can be very reliable in some experiments[22], [44], particularly in the auditory cortex, proving that reliable synapses do exist. So we argue that variability in other recorded spike times, in particular in the visual system, could come from non-controlled variables that might also affect neuronal activation, such as attention, eye movements, mental imagery, top-down effects etc. As Barlow wrote about neural responses in 1972, “their apparently erratic behavior was caused by our ignorance, not the neuron's incompetence.”[47]

We would like to emphasize the fact that the approach presented here is generic. It is not limited to sensory systems, and it could be applied to either experimental or model-generated data. The first step would be to see if STDP finds spike patterns in the data. Providing it does, the second step would be to understand what those patterns mean by solving the corresponding inverse problem.

What happens if there is more than one repeating pattern present in the input? We verified that as the learning progresses, the increasing selectivity of the postsynaptic neuron rapidly prevents it from responding to several patterns. Instead, it picks one (chance determines which one), and becomes selective to it and only to it. To learn the other patterns other neurons are needed.

A competitive mechanism could ensure they optimally cover all the different patterns and avoid learning the same ones. Such a mechanism could be implemented through inhibitory horizontal connections between neurons, such that as soon as one neuron fires, it could prevent other cells from learning the same pattern, as in previous work[48]. The neural population would then self-organize to cover all the input patterns. The ‘coverage’ could be optimized using neurons that differ in their parameters (for example their thresholds), leading to more robust learning and detection. Furthermore a long input pattern can be coded by the successive firings of several STDP neurons, each selective to a different part of the pattern, and competition would prevent them all from tracking back through the pattern and clustering at the beginning. Note that within such a competitive framework a pattern detection probability of 50% is hardly a disaster: it means that with 2 neurons the risk that one pattern is not detected is 25%, with 3 neurons 12.5%, with 4 neurons 6.25% and so on. The system could then work with suboptimal parameters (highlighted in Fig. 7), for example weaker initial weights.

Further work is needed to evaluate this form of competitive network. However in this paper we wanted to stress the fact that *one* single LIF neuron equipped with STDP is consistently able to detect *one* arbitrary repeating spatio-temporal spike pattern embedded in equally dense ‘distractor’ spike trains, which is a remarkable demonstration of the potential for such a scheme.

## Materials and Methods

The simulations were performed using MATLAB R14 (Mathworks 2005, Natick MA). The source code is available from the authors upon request.

### Poisson spike trains

The spike trains were prepared before the simulation (Fig. 1 illustrates the type of spike trains we used, though with a smaller set of neurons). For memory issues instead of using spike trains defined over a 450 seconds period, we pasted the same 150s long pattern three times (this repetition had no impact on the results). Each afferent emits spikes independently using a Poisson process with a variable instantaneous firing rate r, that varies randomly between 0 and 90 Hz. The maximal rate change s was chosen so that the neuron could go from 0 to 90 Hz in 50 ms. To be precise, time was discretized using a time step dt of 1 ms. At each time step:

1. the afferent has a probability of r.dt of emitting a spike (whose exact date is then picked randomly in the 1 ms time bin)
2. its instantaneous firing rate is modified: dr = s.dt where s is the speed of rate change (in Hz/s), and clipped in [0, 90] Hz.
3. its speed of rate change is modified by ds, randomly picked from a uniform distribution over [−360+360] Hz/s, and clipped in [−1800+1800] Hz/s

Note that we chose to apply the random change to s as opposed to r so as to have a continuous s function and a smoother r function.

As mentioned in the Discussion, a limitation of this work is the excitatory-only scheme. Consequently, something like ‘afferent A must not spike’ cannot be learnt, only ‘positive patterns’ can. We thus wanted a pattern in which all the afferents spike at least once. We could have made up such a pattern, but we wanted the pattern to have exactly the same statistics as the Poisson distractor part (to make the pattern detection harder), so we preferred to randomly pick a 50 ms period of the original Poisson spike trains and to ‘copy-paste’ it (see below). To make sure this randomly selected period did contain a spike from each afferent we implemented a mechanism that triggers a spike whenever an afferent has been silent for more than 50 ms (leading to a minimal firing rate of 20 Hz). Clearly, such mechanism is NOT implemented in the brain. It is just an artifice we used here to make the pattern detection harder. As a result the average firing rate was 54 Hz, and not the 45 Hz we would have without this additional mechanism.

Once the random spike train has been generated, a part of it, defined as the ‘pattern’ to be repeated, is ‘copy-pasted’. This ‘copy-paste’ does not involve the last 1000 afferents (obviously the indices are arbitrary), which conserve their original spike trains. But we discretize the spike trains of the first 1000 afferents into 50 ms sections. We randomly pick one of these sections and copy the corresponding spikes. Then we randomly pick a certain number of these sections (1/4 in the baseline condition), avoiding consecutive ones, and replace the original spikes by the copied ones. A jitter was added before the pasting operation, picked from a Gaussian distribution with mean zero and standard deviation 1 ms (in the baseline condition).

After this ‘copy-paste’ operation a 10 Hz Poissonian spontaneous activity was added, to all neurons and all the time. The total activity was thus 64 Hz on average, and spontaneous activity represented about 16% of it.

### Leaky Integrate and Fire (LIF) neuron (see Fig. 3)

For computational reasons we modeled the LIF neuron using Gerstner's Spike Response Model (SRM)[16], [49]. That is instead of solving the membrane potential differential equation we used kernels to model the effect of presynaptic and postsynaptic spikes on the membrane potential. Each presynaptic spike *j*, with arrival time *t_j_*, is supposed to add to the membrane potential an Excitatory Post-Synaptic Potential (EPSP) of the form:
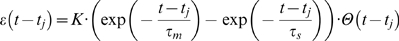
where *τ_m_* is the membrane time constant (here 10 ms), *τ_s_* is the synapse time constant (here 2.5 ms), Θ is the Heavyside step function:
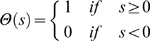
and *K* is just a multiplicative constant chosen so that the maximum value of the kernel is 1 (the voltage scale is arbitrary in this paper).

The last emitted postsynaptic spike *i* has an effect on the membrane potential modeled as follows:
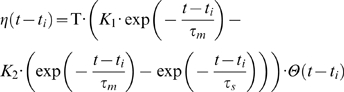
where T is the threshold of the neuron (here 500, arbitrary units). The first term models the positive pulse and the second one the negative spike-afterpotential that follows the pulse (see Fig. 3). Here we used *K*_1_ = 2 and *K*_2_ = 4. For simplicity, the resting potential is supposed to be zero, but a non zero value would simply shift the kernel, and shifting the threshold by the same value would lead to the same computation.

Both *ε* and *η* kernels were rounded to zero when respectively *t*−*t_j_* and *t*−*t_i_* were greater than 7·τ*_m_*.

At any time the membrane potential is:
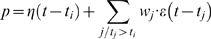
where the *w_j_* are the excitatory synaptic weights, between 0 and 1 (arbitrary units).

This SRM formulation allows us to use event-driven programming: we only compute the potential when a new presynaptic spike is integrated. We then estimate numerically if the corresponding EPSP will cause the threshold to be reached in the future and at what date. If it is the case, a postsynaptic spike is scheduled. Such postsynaptic spike events cause all the EPSPs to be flushed, and a new *t_i_* is used for the *η* kernel. There is then a refractory period of 1 ms, during which the neuron is not allowed to fire.

### Spike Timing Dependent Plasticity

An exponential update rule (see Fig. 2):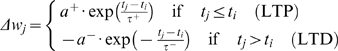 with the time constants *τ* ^+^ = 16.8 ms and *τ* ^−^ = 33.7 ms, provides a reasonable approximation of the synaptic modification observed experimentally[13].We restricted the learning window to [*t_i_*−7·*τ* ^+^,*t_i_*] for LTP and to [*t_i_*,*t_i_*+7·*τ* ^−^] for LTD. For each afferent, we also limited LTP (respectively LTD) to the last (first) presynaptic spike before (after) the postsynaptic one (‘nearest spike’ approximation). We did not take the effects of finer triplet of spikes[50] into account.

It was found that small learning rates led to more robust learning. We used *a* ^+^ = 0.03125 and *a* ^−^ = 0.85·*a* ^+^ Following learning the weights were clipped to [0,1]. Note that all synapses remain excitatory: there is no inhibition in all these simulations.
